# Prognostic Significance of Preoperative Albumin to Alkaline Phosphatase Ratio in Patients with Glioblastoma

**DOI:** 10.7150/jca.61866

**Published:** 2021-08-13

**Authors:** Junhong Li, Mingrong Zuo, Xingwang Zhou, Yufan Xiang, Shuxin Zhang, Wentao Feng, Yanhui Liu

**Affiliations:** Department of Neurosurgery, West China Hospital of Sichuan University, Chengdu 610041, Sichuan Province, P.R. China.

**Keywords:** Albumin to alkaline phosphatase ratio, glioblastoma, prognosis, propensity score matching

## Abstract

**Objective:** To explore the prognostic value of preoperative albumin to alkaline phosphatase ratio (AAPR) in patients with newly-diagnosed glioblastoma (GBM) and its association with clinical characteristics.

**Patients and methods:** A retrospective analysis was carried out on patients with newly diagnosed GBM who had undergone operation at the Department of Neurosurgery at West China Hospital between June 1st 2016 to December 31st 2018. X-tile software was applied to determine the optimal cut-off values for AAPR, neutrophil to lymphocyte ratio (NLR), and albumin. Cox regression analyses were applied to evaluate the prognostic value of AAPR in GBM. PSM analysis was conducted to verify the results.

**Results:** A total of 197 and 154 GBM patients were included in original cohort and PSM cohort respectively. The optimal cut-off value for AAPR, NLR, and albumin were 0.56, 4.55 and 42.2 g/L respectively. High AAPR was only significantly related to longer overall survival (OS) (p=0.010) in original cohort. In PSM cohort, no clinical variable was evidently related to the level of AAPR. AAPR was determined to be an independent prognostic indicator in both original cohort (HR=0.599, 95%CI 0.437-0.822, p=0.001) and PSM cohort (HR=0.649, 95%CI 0.459-0.918, p=0.015). Prognostic models including AAPR had better prognostic accuracy than that including albumin.

**Conclusion:** Preoperative AAPR was determined to be an independent risk factor of prognosis in newly-diagnosed GBM patients, and its prognostic ability was stronger than albumin. And PSM analysis also validated the results.

## Introduction

Glioblastoma (GBM) is the most lethal diffuse glioma with a median overall survival of 14-17 months and is characterized histologically by marked cellularity, prominent mitotic activity, abundant vascular proliferation and necrosis 1, 2. GBM is recognized as grade IV tumor by the World Health Organization (WHO), constituting 45.2% of all malignant central nervous system (CNS) tumors and 80% of all primary malignant CNS tumors 3. Based on the latest 2016 WHO classification of CNS tumors, GBMs are divided into three types, including IDH-wildtype, IDH-mutant, and not otherwise specified (NOS) glioblastoma 4. Surgery is the optimal choice in patients with suspected malignant glioma, and postoperative radiotherapy and chemotherapy are considered to be the first-line adjuvant treatments, which can help prolong lifetime to the maximum extent 1. In recent years, the biological nature of GBM has been well comprehended by deep DNA and RNA sequencing, coupled with improved techniques that enable richer interrogation of the epigenome 5. At the same time, cheaper and more convenient methods including neuroimaging, clinical examination, histopathology, etc. are also contributing to a well-rounded understanding of GBM.

Nowadays, cumulative evidence has indicated that some blood parameters are related to tumorigenesis and tumor progression 6-8. Several biomarkers such as neutrophil-to-lymphocyte ratio (NLR), platelet-to-lymphocyte ratio (PLR), and fibrinogen are considered to be associated with clinical outcomes 9-12. Albumin is one of the important compositions of the blood and constitutes 50% of the plasma. It is responsible for 75% of the plasma oncotic pressure and increases circulating blood volume. Albumin performs a variety of physiological functions, including ligand binding and drugs transport, free radical scavenging, anti-oxidant function, effect on vascular permeability, etc. 13. The hydrolase enzyme alkaline phosphatase (ALP) is widely expressed in human tissues such as liver, bones and kidneys 14. Similarly, it plays an important role in human physiological functions, including bone mineralization, vascular calcification, and regulations of immune system 15-17. The albumin to alkaline phosphatase ratio (AAPR), based on serum albumin and alkaline phosphatase, is a useful prognostic indicator. It has been found to have significant impacts on survival in patients with liver cancer 18 and predictive ability on prognosis in various tumors 19-22.

The relationship between AAPR and GBM has not been explored so far. In the current study, we attempted to figure out the prognostic value of preoperative AAPR in patients with GBM, and investigated its association with clinical characteristics.

## Patients and Methods

### Patient Population

A retrospective chart review was carried out on patients with newly diagnosed GBM who had undergone an operation at the Department of Neurosurgery at West China Hospital between June 1^st^ 2016 to December 31^st^ 2018. All patients underwent a craniotomy on GBM with gross total resection (GTR) or subtotal resection (STR). These patients were followed up until June 31^st^ 2020. The pathological diagnoses were based on 2016 WHO classification of CNS tumors.

The inclusion criteria included: 1) older than 18-year-old; 2) underwent resection of GBM by craniotomy with GTR or STR; 3) the pathological diagnoses were based on the latest 2016 WHO classification; 4) intact baseline clinical data; 5) intact preoperative MRI imaging data and postoperative imaging data including MRI and CT within 72 hours after operation; 6) no adjuvant therapy like chemotherapy or radiotherapy before operation; 7) no history of diseases dramatically affecting peripheral blood cells. The exclusion criteria were: 1) younger than 18-year-old; 2) biopsy only; 3) absence of definite pathological diagnosis; 4) incomplete baseline clinical data; 5) absence of preoperative MRI imaging data; 6) receiving adjuvant therapy before operation; 7) presence of history of liver diseases, bone diseases, urological diseases, or infectious diseases shortly before surgery.

### Parameters Assessment

Medical records were surveyed and the following clinical data were retrieved: gender, age at operation, preoperative Karnofsky performance status, presence of preoperative seizures, locations and hemisphere of tumors, pathological diagnoses and useful biomarkers. Ki-67 index was tested by immunohistochemistry (IHC), and IDH-1 mutations were determined by both IHC and molecular testing. Routine blood tests were performed within 3 days prior to surgery and relevant data was recorded. The APPR was defined as the ratio between the serum albumin concentration (g/L) and the alkaline phosphatase (U/L), while the NLR was defined by dividing the neutrophil (×10^9^/L) count by the lymphocyte count (×10^9^/L).

Postoperative adjuvant therapies and survival conditions were collected mainly through periodical telephone interview and outpatient follow-up. Patients were routinely followed up every 3 months for the first year, and every 6 months thereafter. Overall survival was defined as the duration from the date of surgery to death or the last follow up. All clinical assessments were performed by two independent qualified neurosurgeons.

### Statistical Analysis

SPSS 22.0 (IBM Co., Armonk, NY, USA) was used for all statistical analyses. X-tile software was applied to determine the optimal cut-off values for AAPR, NLR, and albumin 23. The associations between APPR and clinical variables were tested by chi-square test, Mann-Whitney test, or one-way ANOVA (one-factor analysis of variance). The Cox regression analyses were used to determine the influence of risk factors for overall survival in GBM patients. In Cox regression analyses, a univariate Cox regression was firstly conducted to evaluate clinical variables, then variables with p value <0.1 were included into a backward stepwise multivariate Cox regression for further assessment. R software (version 3.6.3, http://www.r-project.org/) was applied to calculate and compare Harrell concordance index (C-index) and Akaike information criterion (AIC) of prognostic models. A smaller AIC value and/or a larger C-index represented a greater predictive accuracy. A two-sided p-value <0.05 referred as statistically significant difference.

Propensity score matching (PSM) analysis was introduced in the current study to adjust for confounding variables and validate the results of the original cohort. The potential confounding covariables included age at diagnosis, gender, preoperative seizures, KPS, hemisphere, location, Ki-67 index, and IDH-1 status. These patients were matched 1:1 using the nearest-neighbor algorithm with a caliper width of 0.2 and without replacement.

### Ethics

This study was approved by the Ethical Committee of Sichuan University and conducted according to the principles expressed in the Declaration of Helsinki, and all patients were informed and signed their informed consent to use their data for research purposes.

## Results

### Baseline Characteristics

**Figure 1:**
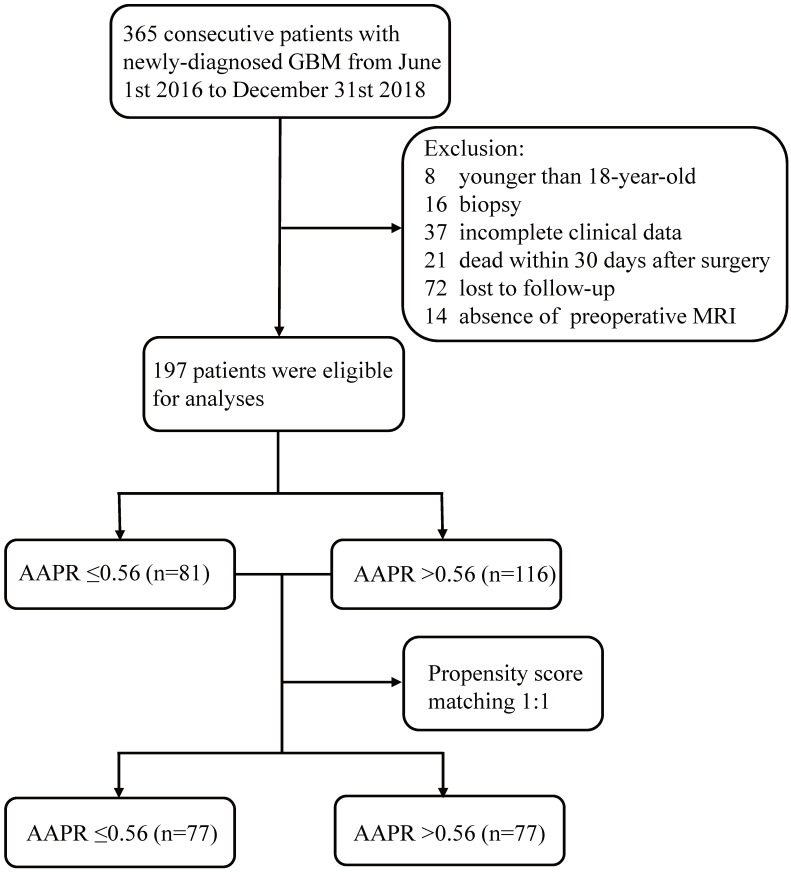
Flow chart of the current study. Abbreviation: GBM, glioblastoma; MRI, magnetic resonance imaging; AAPR, albumin to alkaline phosphatase ratio.

**Figure 2:**
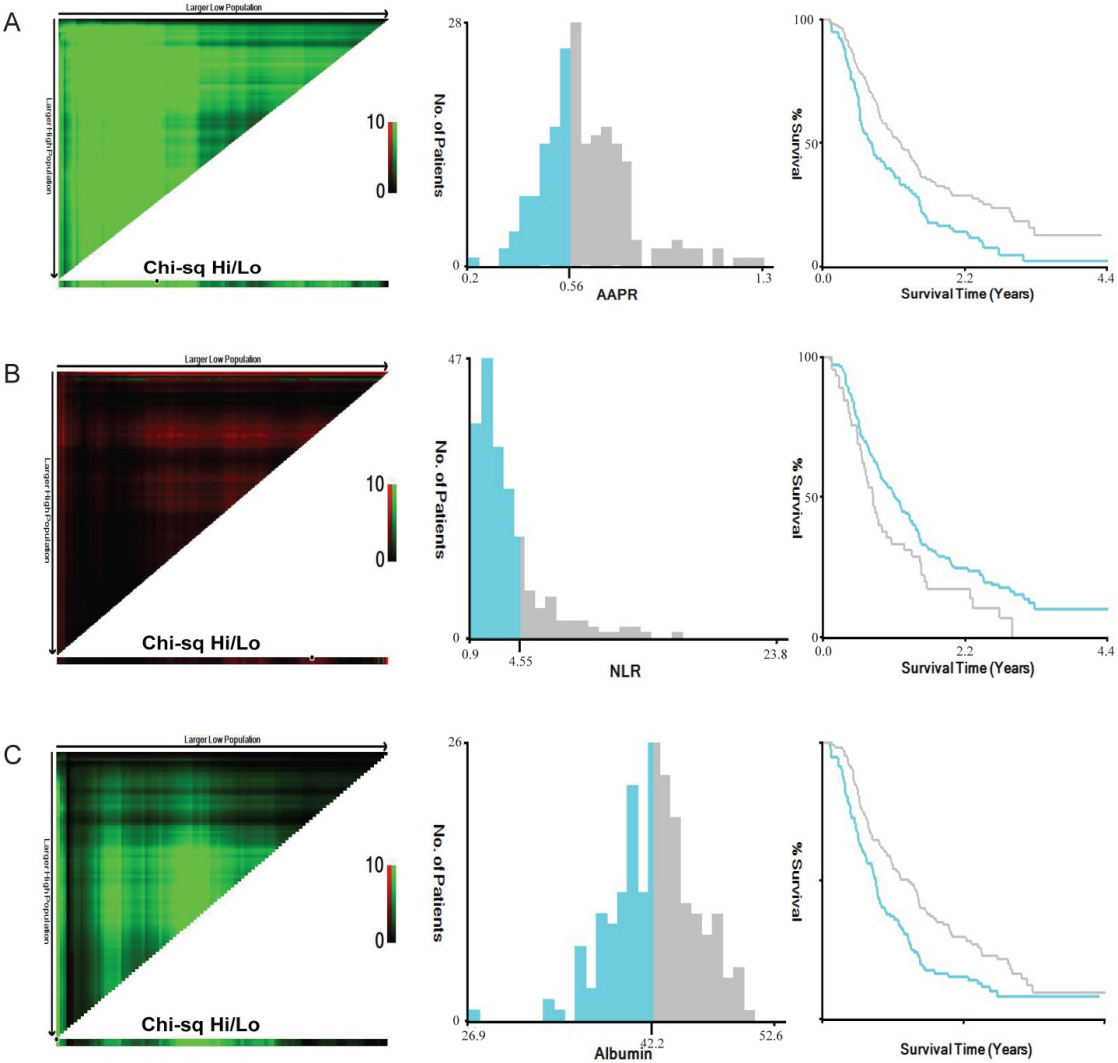
Calculation of optimal cut-off values of AAPR (A), NLR (B) and albumin (C) by X-tile software. Abbreviation: AAPR, albumin to alkaline phosphatase ratio; NLR, neutrophil to lymphocyte ratio.

The screening process was listed in Figure 1. The original cohort was constituted by a total of 197 patients with craniotomy for histologically-proven glioblastoma (Table 1). There were 120 (60.9%) males and 77 (39.1%) females, with a mean age of 54.58 ±0.975 years (median 55, range 20-85 years). The mean follow-up period was 467.06 ±24.57 days (median 357, range 35-1611 days). In terms of tumor-related seizures, 32 (16.2%) patients were diagnosed with preoperative seizures. A preoperative KPS score >80 was recorded in 61 (31.0%) patients and vice versa. For location of GBMs, 95 (48.2%) were located at left hemisphere, 91 (46.2%) at right hemisphere, and 11 (5.6%) at midline regions or invading bilateral brain tissues. These tumors were distributed in frontal lobe (22.84%), temporal lobe (14.21%), parietal lobe (4.1%), and occipital lobe (1.0%), and the rest were involved in multiple regions (57.9%). As for postoperative adjuvant therapy, 69 (35.0%) patients received both chemotherapy and radiotherapy, 59 (30.0%) patients received one of the two treatments, while other 69 (35.0%) patients didn't receive any kind of adjuvant therapy. Specific tumor-related biomarkers were recorded; a total of 104 (52.8%) patients had ki-67 index <30%, while 31 patients were tested as IDH-1 mutation. The optimal cut-off value for AAPR, NLR, and albumin were 0.56, 4.55, and 42.2 g/L calculating by the X-tile software, respectively (Figure 2). An AAPR>0.56 was found in 116 (58.9%) patients, and an NLR>4.55 was shown in 45 (22.8%) patients.

After 1:1 PSM stratified by the optimal cut-off value of AAPR, a total of 154 patients with 77 AAPR>0.56 and 77 AAPR≤0.56 were included in PSM cohort. This cohort had a high similarity to original cohort in constitution.

### Relationships between AAPR and Clinical Variables

The relationships between AAPR and other clinical variables were shown in Table 2. In original cohort, high AAPR was only significantly related to OS (p=0.010), and there was no significant association between AAPR and other clinical variables. In PSM cohort, however, there were not any clinical variables evidently related to the level of AAPR.

### Prognostic value of AAPR

Univariate and multivariate Cox regressions were performed to further determine the prognostic significance of AAPR. As shown in Table 3, in original cohort, univariate Cox regression indicated high AAPR (HR 0.596, 95%CI 0.439-0.810, p=0.001) and high albumin (HR 0.617, 95%CI 0.454-0.837, p=0.002) were significantly associated with better OS, while high NLR was related to poor prognosis (HR 1.490, 95%CI 1.045-2.125, p=0.028). Other significant variables included age, gender, KPS, hemisphere, adjuvant therapy, ki-67 index, and IDH-1 status.

Variables with a p value<0.1 was included in multivariate Cox regression. AAPR (HR=0.599, 95%CI 0.437-0.822, p=0.001) and albumin (HR=0.670, 95%CI 0.484-0.929, p=0.016) were proved to be independent risk factors of OS. Other independent risk factors included age, gender, adjuvant therapy, and ki-67 index, while hemisphere and NLR were not independent prognostic factors.

As regard to PSM cohort (Table 4), independent prognostic indicators from multivariate Cox regression included adjuvant therapy, IDH-1 status, AAPR, and albumin.

### Comparison of prognostic ability between AAPR and albumin

To further compare the prognostic predictive ability of AAPR and albumin, prognostic models were established by using the two markers combined with other independent variables. In original cohort (Table 5), C-index and AIC were calculated by R software and the results indicated that model AAPR (C-index/AIC, 0.721/1452.24) was superior to model albumin (C-index/AIC, 0.715/1455.95). As the same, in PSM cohort (Table 6), model AAPR (C-index/AIC, 0.698/1091.43) had advantage over model albumin (C-index/AIC, 0.695/1094.86) in predictive accuracy.

## Discussion

Peripheral blood markers were widely used in monitoring tumor progression and predicting prognosis of tumor patients. In the current study, we found higher AAPR was associated with better OS in patients with GBM. Multivariate analysis indicated AAPR as an independent risk factor for OS. And PSM analysis also verified the results.

The results are consistent with the conclusions of other researches about AAPR to some extent. Previous researches report that AAPR is evidently related to the clinical outcome of solid tumors, namely higher AAPR is significantly associated with better outcomes in survival or therapy of the tumors, such as upper tract urothelial carcinoma, cholangiocarcinoma, non-small-cell lung cancer, hepatocellular carcinoma, nasopharyngeal carcinoma, pancreatic ductal adenocarcinoma, etc. 18-20, 22, 24, 25. Cut-off values of these studies were in the range of 0.36-0.84, and the cut-off value of AAPR in our research was 0.56. Due to large sample size, results of these different tumor researches are considered reliable.

Albumin is an important nutritional indicator and also associated with the process of systemic inflammation 26, 27. Previous researches indicate albumin served as a prognostic predictor in various kind of tumors, including oral cavity squamous cell carcinoma, non-small cell lung cancer, advanced esophageal cancer, etc. 28-30. Higher albumin level is often related to better clinical outcome. And to enhance the prediction, it is also combined with other blood biomarkers like Glasgow Prognostic Score (GPS) and Controlling Nutritional Status Score (CONUT) 31, 32. In view of the origin of ALP, the correlations between ALP and prognosis of tumor were firstly reported in osteosarcoma, advanced prostate cancer, and hepatocellular carcinoma 33-35. Then, studies of other tumors have found higher peripheral blood APL indicating shorter overall survival or progression-free survival 36-38. This may explain why albumin to alkaline phosphatase ratio has a predictive ability of prognosis of tumors, but the underlying mechanism remains unclear.

Another hot blood biomarker NLR was introduced in our study to serve as a reference. As a marker of systemic inflammation, NLR is supposed to have predictive ability of prognosis in tumor patients based on the theory that inflammatory response plays an important role in tumor development and progression 11. Most studies indicate that high NLR is related to shorter OS or PFS 9, 10, 39, 40. In the research of NLR and glioma, the situation becomes complicated. Majority of studies show that high NLR is significantly related to bad prognosis in multivariate Cox regression analyses 41-44. Conversely, some researches are unable to show that high NLR predicts shorter survival in GBM 45, 46. Our study indicated that NLR was not an independent prognostic factor for GBM (multivariate Cox regression, HR 1.015, 95%CI 0.696-1.481, p=0.937).

The examinations and treatments of GBM are expensive and represent a significant economic burden on health care system all around the world 47. Compare with liquid biopsy, molecular test, and advanced magnetic resonance imaging (MRI) examination, blood biomarkers such like AAPR, NLR, platelet to lymphocyte ratio (PLR), and lymphocyte to monocyte ratio (LMR) are convenient, cheap, and time-saving. These easily acquired parameters help us understand this disease in more comprehensive perspective and make an accurate judgment on diagnosis and treatment strategies in GBM patients.

There are still some limitations in the current study. At first, the sample size is relatively small so that results of the study could not be further validated using external validation methods. Secondly, postoperative AAPR should be included in the follow-up plan so that we can further evaluate the prognostic value throughout the course of GBM. Thirdly, progression of GBM could not be accurately assessed, some patients didn't even have a chance to be reexamined after operation, so we didn't include this part in our research. Fourthly, this research was just conducted by a single center, and multi-center collaboration should be reached to further verify the results.

## Conclusion

To our knowledge, this is the first study focusing on validating the prognostic ability of preoperative AAPR in GBM. In the current study, AAPR is determined to be an independent risk factor of prognosis in patients with newly-diagnosed GBM, and its prognostic predictive ability is stronger than albumin. PSM analysis is also conducted to validate the results. Serum albumin and ALP are simple, affordable and relatively innocuous test that could serve as an objective prognostic parameter for GBM.

## Figures and Tables

**Table 1:** Baseline clinical characteristics of GBM patients in original cohort and PSM cohort

Clinical Characteristic	Original cohort	PSM cohort
Number of patients	197 (100)	154 (100)
**Follow-up period**		
Mean± SD (day)	467.06 ±344.83	443.62 ±322.78
Median (range)	357 (35-1611)	337 (38-1611)
**Age at operation**		
Mean± SD (year)	54.58 ±13.68	55.46 ±13.11
Median (range)	55 (20-85)	56 (20-85)
**Gender**		
Male	120 (60.9)	100 (64.9)
Female	77 (39.1)	54 (35.1)
**Preoperative seizures**		
Yes	32 (16.2)	26 (16.9)
No	165 (83.8)	128 (83.1)
**KPS**		
>80	61 (31.0)	45 (29.2)
≤80	136 (69.0)	109 (70.8)
**Hemisphere**		
Left	95 (48.2)	73 (47.4)
Right	91 (46.2)	71 (46.1)
Midline or bilateral	11 (5.6)	10 (6.5)
**Location**		
Frontal lobe	45 (22.8)	36 (23.4)
Temporal lobe	28 (14.2)	24 (15.6)
Parietal lobe	8 (4.1)	4 (2.6)
Occipital lobe	2 (1.0)	1 (0.6)
Other locations	114 (57.9)	89 (57.8)
**Adjuvant therapy**		
Chemotherapy and radiotherapy	69 (35.0)	56 (36.4)
Chemotherapy or radiotherapy	59 (30.0)	46 (29.9)
None	69 (35.0)	52 (33.7)
**Ki-67**		
≥30%	93 (47.2)	76 (49.4)
<30%	104 (52.8)	78 (50.6)
**IDH-1**		
Mutant	31 (15.7)	21 (13.6)
Wildtype	166 (84.3)	133 (86.4)
**NLR**		
>4.55	45 (22.8)	38 (24.7)
≤4.55	152 (77.2)	116 (75.3)
**Albumin (g/L)**		
>42.2	105 (53.3)	80 (51.9)
≤42.2	92 (46.7)	74 (48.1)
**AAPR**		
>0.56	116 (58.9)	77 (50.0)
≤0.56	81 (41.1)	77 (50.0)

Data are expressed as n (%), mean ± SD, or median (range); Abbreviations: GBM, glioblastoma; PSM, propensity score matching; KPS, Karnofsky performance status; IDH-1, Isocitrate dehydrogenase-1; NLR, neutrophil to lymphocyte ratio; AAPR, albumin to alkaline phosphatase ratio.

**Table 2:** Relationship between AAPR and clinical characteristics in original cohort and PSM cohort

Clinical Characteristic	Original cohort			PSM cohort		
	AAPR≤0.56 (n=81)	AAPR>0.56 (n=116)	p value	AAPR≤0.56 (n=77)	AAPR>0.56 (n=77)	p value
Age at operation	53.65 ±13.78	55.93 ±13.51	0.166	55.12 ±13.32	55.81 ±12.98	0.884
OS	277 (35-1611)	421 (38-1575)	***0.010***	286 (43-1611)	384 (38-1575)	0.140
**Gender**						
Male	52 (64.2)	68 (58.6)	0.431	49 (63.6)	51 (66.2)	0.736
Female	29 (35.8)	48 (41.4)		28 (36.4)	26 (33.8)	
**Preoperative seizures**						
Yes	15 (18.5)	17 (14.7)	0.471	14 (18.2)	12 (15.6)	0.668
No	66 (81.5)	99 (85.3)		63 (81.8)	65 (84.4)	
**KPS**						
>80	24 (29.6)	37 (31.9)	0.736	23 (29.9)	22 (28.6)	0.860
≤80	57 (70.4)	79 (68.1)		54 (70.1)	55 (71.4)	
**Hemisphere**						
Left	37 (45.7)	58 (50.0)	0.366	35 (45.5)	36 (46.7)	0.630
Right	36 (44.4)	55 (47.4)		35 (45.5)	38 (49.4)	
Midline or bilateral	8 (9.9)	3 (2.6)		7 (9.0)	3 (3.9)	
**Location**						
Frontal lobe	19 (23.4)	26 (22.4)	0.511	18 (23.4)	18 (23.4)	0.785
Temporal lobe	16 (19.8)	12 (10.3)		15 (19.5)	9 (11.7)	
Parietal lobe	1 (1.2)	7 (6.0)		0 (0.0)	4 (5.2)	
Occipital lobe	0 (0.0)	2 (1.7)		0 (0.0)	1 (1.3)	
Other regions	45 (55.6)	69 (59.5)		44 (57.1)	45 (58.4)	
**Ki-67**						
≥30%	42 (51.9)	51 (44.0)	0.276	39 (50.6)	37 (48.1)	0.748
<30%	39 (48.1)	65 (56.0)		38 (49.4)	40 (51.9)	
**IDH-1**						
Mutant	9 (11.1)	22 (19.0)	0.137	9 (11.7)	12 (15.6)	0.483
Wildtype	72 (88.9)	94 (81.0)		68 (88.3)	65 (84.4)	

Data are expressed as n (%), mean ± SD, or median (range). Significant findings are expressed in bold and italic. Abbreviations: AAPR, albumin to alkaline phosphatase ratio; PSM, propensity score matching; KPS, Karnofsky performance status; IDH-1, isocitrate dehydrogenase-1; SD, standard deviation.

**Table 3:** Univariate and multivariate Cox regression for risk factors predictive of GBM in original cohort

	Univariate Analysis				Multivariate Analysis (AAPR)				Multivariate Analysis (Albumin)			
	HR	LL	UL	p value	HR	LL	UL	p value	HR	LL	UL	p value
**Age at operation**												
≥55	1				1				1			
<55	0.566	0.416	0.771	***<0.001***	0.706	0.507	0.983	***0.040***	0.772	0.547	1.088	0.140
**Gender**												
Male	1				1.000				1.000			
Female	0.670	0.488	0.919	***0.013***	0.663	0.472	0.931	***0.018***	0.671	0.480	0.937	***0.019***
**KPS**												
>80	1											
≤80	1.233	0.884	1.721	0.218								
**Hemisphere**												
Midline or bilateral	1				1				1			
Right	0.502	0.259	0.974	***0.041***	0.767	0.387	1.521	0.448	0.748	0.378	1.477	0.403
Left	0.395	0.203	0.769	***0.006***	0.511	0.256	1.021	0.057	0.487	0.245	0.967	***0.040***
**Location**												
Frontal lobe	1											
Temporal lobe	1.639	0.983	2.731	0.058								
Parietal lobe	1.005	0.422	2.395	0.991								
Occipital lobe	0.383	0.052	2.813	0.346								
Other regions	1.319	0.896	1.943	0.160								
**Pre-operative seizures**												
No	1											
Yes	1.242	0.836	1.845	0.284								
**Adjuvant therapy**												
Chemotherapy and radiotherapy	1				1				1			
Chemotherapy or radiotherapy	2.591	1.745	3.847	***<0.001***	3.016	1.998	4.553	***<0.001***	3.205	2.124	4.835	***<0.001***
None	3.506	2.387	5.148	***<0.001***	4.595	2.991	7.059	***<0.001***	4.492	2.951	6.838	***<0.001***
**Ki67**												
≥30%	1				1				1			
<30%	0.736	0.544	0.997	***0.048***	0.638	0.462	0.882	***0.007***	0.681	0.496	0.936	***0.018***
**IDH-1**												
Positive	1				1				1			
Negative	2.765	1.665	4.590	***<0.001***	2.055	1.209	3.492	***0.008***	2.369	1.396	4.021	***0.001***
**NLR**												
Low	1				1				1			
High	1.490	1.045	2.125	***0.028***	1.028	0.706	1.498	0.884	0.985	0.672	1.444	0.938
**AAPR**												
Low	1				1							
High	0.596	0.439	0.810	***0.001***	0.599	0.437	0.822	***0.001***				
**Albumin**												
Low	1								1			
High	0.617	0.454	0.837	***0.002***					0.670	0.484	0.929	***0.016***

Significant findings are expressed in bold and italic. Abbreviations: GBM, glioblastoma; HR, hazard ratio; CI, confidence interval; LL, lower limit; UL, upper limit; KPS, Karnofsky performance status; IDH-1, Isocitrate dehydrogenase-1; NLR, neutrophil to lymphocyte ratio; AAPR, albumin to alkaline phosphatase ratio.

**Table 4:** Univariate and multivariate Cox regression for risk factors predictive of GBM in PSM cohort

	Univariate Analysis				Multivariate Analysis (AAPR)				Multivariate Analysis (Albumin)			
	HR	95% CI		p value	HR	95% CI		p value	HR	95% CI		p value
		LL	UL			LL	UL			LL	UL	
**Age at operation**												
≥55	1				1				1			
<55	0.675	0.477	0.954	***0.025***	0.878	0.613	1.258	0.479	0.984	0.682	1.420	0.932
**Gender**												
Male	1											
Female	0.756	0.524	1.091	0.135								
**KPS**												
>80	1											
≤80	1.013	0.699	1.468	0.947								
**Hemisphere**												
Midline or bilateral	1											
Right	0.580	0.287	1.172	0.129								
Left	0.458	0.226	0.931	***0.031***								
**Location**												
Frontal lobe	1											
Temporal lobe	1.404	0.804	2.453	0.233								
Parietal lobe	0.919	0.280	3.024	0.890								
Occipital lobe												
Other regions	1.248	0.813	1.917	0.311								
**Pre-operative seizures**												
No	1											
Yes	1.104	0.709	1.719	0.660								
**Adjuvant therapy**												
Chemotherapy and radiotherapy	1				1				1			
Chemotherapy or radiotherapy	2.809	1.803	4.375	***<0.001***	2.866	1.834	4.478	***<0.001***	3.119	1.986	4.899	***<0.001***
None	4.085	2.632	6.339	***<0.001***	4.062	2.605	6.335	***<0.001***	3.754	2.410	5.849	***<0.001***
**Ki67**												
≥30%	1											
<30%	0.813	0.579	1.143	0.235								
**IDH-1**												
Positive	1				1				1			
Negative	2.688	1.474	4.901	***0.001***	2.584	1.405	4.752	***0.002***	2.702	1.468	4.976	***0.001***
**NLR**												
Low	1											
High	1.338	0.904	1.981	0.146								
**AAPR**												
Low	1				1							
High	0.679	0.482	0.956	***0.027***	0.649	0.459	0.918	***0.015***				
**Albumin**												
Low	1								1			
High	0.664	0.472	0.935	***0.019***					0.664	0.466	0.947	***0.024***

Significant findings are expressed in bold and italic. Abbreviations: GBM, glioblastoma; PSM, propensity score matching; HR, hazard ratio; CI, confidence interval; LL, lower limit; UL, upper limit; KPS, Karnofsky performance status; IDH-1, Isocitrate dehydrogenase-1; NLR, neutrophil to lymphocyte ratio; AAPR, albumin to alkaline phosphatase ratio.

**Table 5:** Prognostic models included AAPR and albumin for GBM patients in original cohort

Clinical variables	Prognostic model (AAPR)				Prognostic model (albumin)			
	HR	95% CI		p value	HR	95% CI		p value
		LL	UL			LL	UL	
**Gender**								
Male	1				1			
Female	0.649	0.466	0.904	***0.011***	0.662	0.475	0.922	***0.015***
**Adjuvant therapy**								
Chemotherapy and radiotherapy	1				1			
Chemotherapy or radiotherapy	2.763	1.852	4.122	***<0.001***	2.972	1.985	4.452	***<0.001***
None	4.248	2.839	6.356	***<0.001***	4.033	2.699	6.026	***<0.001***
**Ki67**								
≥30%	1				1			
<30%	0.732	0.535	1.002	0.051	0.732	0.534	1.003	0.052
**IDH-1**								
Mutant	1				1			
Wildtype	2.265	1.347	3.809	***0.002***	2.405	1.420	4.074	***0.001***
**AAPR**								
Low	1							
High	0.589	0.430	0.807	***0.001***				
**Albumin**								
Low					1			
High					0.632	0.458	0.873	***0.005***
**C-index**	0.721				0.715			
**AIC**	1452.24				1455.95			

Significant findings are expressed in bold and italic. Abbreviations: HR, hazard ratio; CI, confidence interval; LL, lower limit; UL, upper limit; IDH-1, Isocitrate dehydrogenase-1; AAPR, albumin to alkaline phosphatase ratio; GBM, glioblastoma.

**Table 6:** Prognostic models included AAPR and albumin for GBM patients in PSM cohort

Clinical variables	Prognostic model (AAPR)				Prognostic model (Albumin)			
	HR	95% CI		p value	HR	95% CI		p value
		LL	UL			LL	UL	
**Adjuvant therapy**								
Chemotherapy and radiotherapy	1				1			
Chemotherapy or radiotherapy	2.866	1.834	4.478	***<0.001***	3.119	1.986	4.899	***<0.001***
None	4.062	2.605	6.335	***<0.001***	3.754	2.410	5.849	***<0.001***
**IDH-1**								
Mutant	1				1			
Wildtype	2.584	1.405	4.752	***0.002***	2.702	1.468	4.976	***0.001***
**AAPR**								
Low	1							
High	0.649	0.459	0.918	***0.015***				
**Albumin**								
Low					1			
High					1.505	1.056	2.145	***0.024***
C-index	0.698				0.695			
AIC	1091.43				1094.86			

Significant findings are expressed in bold and italic. Abbreviations: PSM, propensity score matching; HR, hazard ratio; CI, confidence interval; LL, lower limit; UL, upper limit; IDH-1, Isocitrate dehydrogenase-1; AAPR, albumin to alkaline phosphatase ratio; GBM, glioblastoma.
